# Three-dimensional assessment of pharyngeal airway in individuals with myotonic dystrophy type 1

**DOI:** 10.3906/sag-2105-106

**Published:** 2021-09-30

**Authors:** Burcu EVLİCE, Filiz KOÇ, Hazal DUYAN, Damla SOYDAN ÇABUK

**Affiliations:** 1 Department of Oral and Maxillofacial Radiology, Faculty of Dentistry, Çukurova University, Adana, Turkey; 2 Department of Neurology, Faculty of Medicine, Çukurova University, Adana, Turkey

**Keywords:** Cone beam computed tomography, myotonic dystrophy type 1, pharyngeal airway, pulmonary function

## Abstract

**Background/aim:**

The objectives of this study were to assess pharyngeal airway volume (PAV) in patients with myotonic dystrophy type 1 (DM1) by cone-beam computerized tomography (CBCT) and to evaluate the impact of diaphragm thickness and pulmonary function tests on PAV.

**Materials and methods:**

Thirty DM1 patients (10 female and 20 male; mean age 42.40 ± 12.07) were included in the study. Age and sex-matched thirty patients were participated as control group. In DM1 group pulmonary function tests (PFT) were performed. Independent *t*-test was used to compare PAV values of patients with DM1 and control group. The Mann–Whitney *U* test was used to compare the parameters according to sex (*p <* 0.05). Pearson and Spearman correlation tests were used to evaluate the relationships between parameters of DM1 patients (*p <* 0.05). A multiple linear regression analysis was performed to explain the PAV with parameters that showed positive correlation with PAV.

**Results:**

Age of onset and disease duration were 22.37 ± 8.45 and 20.03 ± 12.08, respectively, in patients with DM1. PAV values of control group were significantly lower than DM1 group (*p <* 0.001). Forced expiratory volume in 1 s and forced volume vital capacity values were higher in males than females in DM1 group according to sex (*p <* 0.001). PAV values were greater in male patients than females of the DM1 group (*p =* 0.022). Diaphragm thickness in DM1 group after inspiration and expiration were 2.60 ± 0.65 and 1.94 ± 0.40, respectively. According to the regression analysis, DTai and FVC were significantly explained the PAV.

**Conclusion:**

PAV was higher in DM1 group. There was a significant positive correlation between diaphragm thickness, pulmonary functions, and PAVs of DM1 patients. The amount of the PAV was mostly influenced by DTai and FVC. It is recommended to evaluate the PAV in patients with DM1 because of impaired respiratory functions and pharyngeal muscle involvement.

## 1. Introduction

Myotonic dystrophy type 1 (DM1) inherited as an autosomal dominant is a dynamic trinucleotide repeat disease [1]. In DM1, which is a progressive and pleiotropic disease, there is an unstable (CTG) reexpansion in the 3 ‘untranslated region of the myotonic dystrophy protein kinase (DMPK) gene on chromosome 19q13.3 [1]. The prevalence of the disease, which is the most common type of muscular dystrophy in adult age, is 0.5–18.1 / 100.000 [2]. Clinical findings are characterized by multisystemic findings such as muscle weakness, masticatory atrophy, dysphagia, endocrinopathies, cardiac conduction defects, cataracts, cholelithiasis, cognitive impairment, and respiratory failure [3,4]. Reduced muscle strength and motor function in a group of patients with DM1 was shown by Kroksmark et al. [5].

Respiratory failure is one of the important factors determining prognosis of DM1 [6]. During the follow-up period, the respiratory system should be evaluated at regular intervals [6]. Alveolar hypoventilation of respiratory muscles may cause weakness of the pharyngeal muscles, which impact on both respiratory and swallowing function [7–9]. Chewing / facial growth pattern, obstructive sleep apnea syndrome (OSAS), and pharyngeal airway volume (PAV) are closely related to pharynx morphology [10]. Various studies in patients with DM1 have evaluated muscular function, respiratory function, and cognitive dysfunction [11–13]. However, no study has assessed pharyngeal airways of patients with DM1.

In the present study, taking into account the involvement of the pharyngeal muscles, it was aimed to measure the pharyngeal airway volume (PAV) in patients with DM1 with cone-beam computed tomography (CBCT) and to evaluate the impact of diaphragm thickness (DTai: diaphragm thickness after inspiration, DTae: diaphragm thickness after expiration) and pulmonary function tests ((PFT) (FEV1: forced expiratory volume in 1 s and FVC: forced volume vital capacity) on this parameter [14].

## 2. Materials and methods

### 2.1. Study population

This study was conducted in accordance with the principles of the 1964 Helsinki Declaration and was approved by the Non-Interventional Clinical Research Ethics Committee of Çukurova University Faculty of Medicine (Protocol no: 2020 / 95-45). A total of sixty adult individuals were included in the present study. CBCT images of 30 patients (20 males and 10 females) with DM1 and age- and sex-matched 30 healthy controls (20 males and 10 females) were retrospectively evaluated from the archives of the Oral and Maxillofacial Radiology Department of Çukurova University. CBCT scans of both DM1 patients and the control group were previously performed for various dental reasons, so they were already available in the archive of Department of Oral and Maxillofacial Radiology. The control group included the individuals with height and weight records, and, without tracheal and lung diseases that affect the airway, any systemic disease or use of any drugs that may affect respiratory parameters. The standard protocol at Oral and Maxillofacial Radiology Department contains acquiring signed informed consent from all patients for evaluation of their CBCT records for any reasons.

### 2.2. DM1 patients

The patients included to the study were diagnosed with DM1 based on molecular studies at the Neuromuscular Diseases outpatient clinic of Çukurova University Faculty of Medicine, Department of Neurology. Up-to-date information and previous test results of the patients received from the supervisor of Neuromuscular Diseases clinic. Age, sex, body mass index (BMI), age of onset (AO), and disease duration (DD) of the patients were recorded. Pulmonary function tests (PFT) were performed with CareFusion micro RPM device. Diaphragm thickness was measured in B-mode ultrasonography (USG) (GE, logiq P6) with a high frequency (8–12 mHz) using linear transducer at the end of the inspiration and expiration. The thickness was measured from the two echogenic lines (peritoneal line and pleural line).

### 2.3. Imaging procedure

The maxillofacial imaging of the patients was performed with CBCT device (Planmeca ProMax 3D Mid, Helsinki, Finland; exposure parameters: 90 kV, 10 mA, 27 sec scan time, voxel size: 0.4 mm^3^) in Çukurova University Faculty of Dentistry, Department of Oral and Maxillofacial Radiology. As a standard CBCT imaging protocol, the patients’ heads were positioned parallel to the horizontal plane of the Frankfurt plane. The CBCT scans were acquired while the patients closed their teeth in maximum intercuspation. Images were evaluated using a 22-inch LG Flatron monitor (LG, Seoul, Korea) set at a screen resolution of 1440 × 900 pixel and 32-bit color depth.

### 2.4. Image analysis

**Figure 1:**
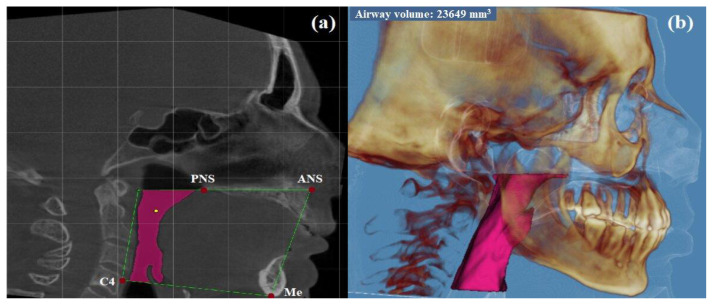
Evaluation of pharyngeal airway volume in sagittal CBCT images (a) Determination of pharyngeal airway boundaries in CBCT’s sagittal section. (b) 3D imaging of the pharyngeal airway volume CBCT, cone beam computed tomography; ANS: anterior nasal spina; PNS: posterior nasal spina; C4: supero-anterior edge of fourth cervical vertebra; Me: menton.

Images in DICOM format were transferred to Dolphin 3D software (version 11.95; Dolphin Imaging & Management Solutions, Chatsworth, California). According to Anandarajah et al., the upper limit according to the pharyngeal airway boundaries was defined as the line passing through the anterior nasal spina (ANS) and the posterior nasal spina (PNS) points [15]. The lower border was defined as the line passing through the supero-anterior edge of the fourth cervical vertebra (C4) and the mentone (Me) points. The anterior border was defined as the anterior wall of the pharynx. The posterior border was defined as posterior wall of the pharynx and the lateral borders were defined as lateral pharyngeal walls. The most appropriate threshold value for each patient was determined to cover the maximum area by manually controlling the sagittal, coronal, and axial sections, respectively, and then the software automatically calculated the PAV (Figure 1a–1b). Data were compared with the data of 30 age and sex-matched healthy control group patients.

### 2.5. Statistical analysis

Before the statistical analysis, the Kolmogorov–Smirnov normality test was used to determine whether the data were normally distributed. Independent *t*-test was used to compare PAV values of patients with DM1 and control group. The Mann–Whitney *U* test was used to compare the parameters according to sex (*p <* 0.05). Pearson and Spearman correlation tests were used to evaluate the relationships between parameters of DM1 patients (*p <* 0.05). A multiple linear regression analysis was performed to explain the PAV with parameters that showed positive correlation with PAV. Radiological PAV measurements were performed twice by two examiners (BE, HD), two weeks apart and independently. Interrater reliability was calculated with the intraclass correlation coefficient (ICC). Pearson correlation coefficient (CC) was used for intra-rater reliability. The ICCs and Pearson CCs were greater than 0.90 for each comparison (*p <* 0.01). In this way, the calibration had been verified for PAV. Mean values and standard deviations for parametric distributed data, median and minimum-maximum values for nonparametric distributed data are shown in the tables. IBM SPSS software version 25.0 (IBM Corp., Armonk, NY) statistical program was used for statistical analysis.

## 3. Results

A total of 60 individuals, 30 DM1 (10 female /20 male) patients with a mean age of 42.40 ± 12.07 were included in the study. Thirty patients who were age and sex-matched were included in the study as control group.

AO and DD were 22.37 ± 8.45 and 20.03 ± 12.08; the measurements of DTai and DTae were 2.60 ± 0.65 and 1.94 ± 0.40; FEV1 and FVC were 2.77 ± 1.22 and 3.33 ± 1.29 respectively, in patients with DM1 (Table 1).

The BMIs (*p =* 0.023) of the control group were significantly higher than the DM1 group (*p <* 0.001), and the PAV values were lower (*p <* 0.001) (Table 1).

FEV1 and FVC values were higher in males than females in DM1 group in terms of sex (*p <* 0.001). While the PAV values of the DM1 group were greater in male patients than in female patients (*p =* 0.022), no significant difference was found in the control group (Table 2).

A positive correlation was found between the DTai, DTae, FEV1 and FVC values of DM1 patients and their PAV values (*p <* 0.05) (Table 3). There was no correlation between PAV and AO, DD, and BMI (Table 3).

Multiple linear regression analysis was performed to develop a model that elucidates the PAV with parameters (DTai, DTae, FEV1 ve FVC) that showed positive correlation with PAV. The total variance of the PAV explained by the regression model [*F*(4, 25) = 23.130, *p <* 0.001)] was 75.3% (R^2^_adjusted_ was 0.753).

According to the analysis, DTai (*β* = 0.311, *t* (25) = 2.620, *p =* 0.015, *pr^2^* = 0.215) and FVC (*β* = 0.641, *t* (25) = 4.185, *p <* 0.001, *pr^2^* = 0.412) were significantly explained the PAV. PAV was not significantly influenced by the DTae and FEV1 according to regression model.

**Figure 2:**
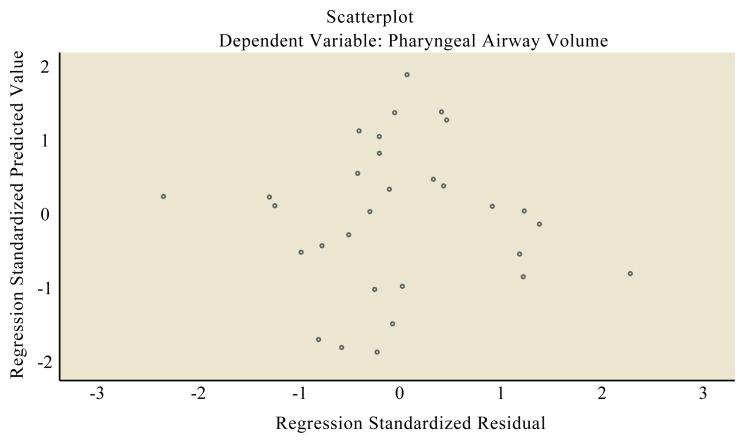
The predicted equation: PAV= −11417.25 + 4006.49*DTai + 3174.31*DTae − 762.73*FEV1 + 7836.55*FVC. DTae: Diaphragm thickness after expiration. DTai: Diaphragm thickness after inspiration. FEV1: Forced expiratory volume in 1 second. FVC: Forced volume vital capacity. PAV: Pharyngeal airway volume.

The predicted equation was as follows:


$$\text{PAV}=-11417.25+4006.49^{★}\text{DTai}+3174.31^{★}\text{DTae}-762.73^{★}\text{FEVI}+7836.55^{★}\text{FVC}$$(
Figure 2).

## 4. Discussion

Respiratory dysfunction is one of the most important symptoms in DM1, which is the most common muscular dystrophy in adults with multiple systemic involvements [16,17]. In a recent study by Rossi et al., the restrictive pulmonary disease was found in 51.9% of 251 DM1 patients [18]. They also stated that half of them needed a non-invasive mechanical ventilator [18]. Various studies were reporting that the causes of mortality in DM1 patients are significantly related to respiratory problems [19–21]. One of the most important parts of the respiratory system is the upper respiratory tract. Anatomical structures such as skeletal structures, muscles surrounding the pharynx, and soft tissues determine the size and configuration of the upper respiratory tract [10,22]. Chewing / facial growth pattern, obstructive sleep apnea syndrome (OSAS), and PAV are closely related to pharynx morphology [10].

Despite greater hyoid bone excursion, patients with DM1 generally have pharyngeal residue, and it often takes a prolonged period of time to clear this residue from the pharynx [23,24]. Decreased pressures in the tongue and pharynx were observed in patients with DM1 [24]. Umemoto et al. have suggested that deterioration of the masticatory and lingual muscles in patients with DM1 completely impairs oropharyngeal function [24].

The present study focused on the evaluation of PAVs of DM1 patients and detecting the impact of diaphragm thickness and PFTs on PAV. It is thought that the evaluation of the pharyngeal airway will be beneficial for clinicians due to impaired respiratory functions and pharyngeal muscle involvement in DM1 patients.

Generally, lateral cephalogram was used in the studies evaluating the pharyngeal airway [25]. However, the cephalometric technique, which has various disadvantages such as low repeatability, distortion, superposition, and magnification due to landmark identification problems [25] is not a suitable method for volume assessment since it is a two-dimensional imaging method [26]. With CBCT, which is a three-dimensional imaging method, the oral and maxillofacial complex has been evaluated in detail with many sections [27–29]. CBCT attracted researchers because of its accurate 3D reconstruction and high image quality presentation [30]. In addition, CBCT has a much lower radiation dose than and faster scanning time compared to computed tomography (CT) [27]. It had been considered an acceptable, effective, and simple imaging method for PAV measurement [22,31,32].

Increased PAV in DM1 may manifest as an adaptation in response to impaired respiratory functions and diaphragm thickness [33]. It is known that pharyngeal airway volumes increase in OSAS patients, especially with mandibular advancement operations [33]. In the present study, PAV values of DM1 patients were significantly higher than the control group (*p <* 0.001).

Respiratory problems are one of the major problems in DM1 patients [34]. Although the prevalence of sleep apnea has been reported between 15.8% and 75%, to the best of our knowledge, there were no studies examining airway volumes in DM1 patients [33–35]. Although there was no statistically significant difference in PAV values between sexes in the control group, PAV was significantly higher in male patients in the DM1 group (*p =* 0.022). It was thought that this may be due to the fact that pharyngeal airways in male patients in the DM1 group show more adaptive changes to impaired respiratory functions. This situation can be clarified by further studies examining the relationship between airways and other factors that may be predisposing.

The diaphragm is the main inspiratory pump muscle and works together with the pharyngeal respiratory muscles to maintain airway patency [36]. USG which has many advantages such as lack of ionizing radiation, ease of application, high spatial resolution, and dynamic imaging, can be used to measure diaphragm thickness in neuromuscular and pulmonary diseases [37,38]. Therefore, it was also used to evaluate the diaphragm muscles of the patients included in the present study. In previous studies, it was reported that the diaphragm thickness measured by USG was significantly lower in patients with DM1 [39,40].

One of the indicators of lung functions is considered to be PFT (FEV1, FVC) values [41]. While Henke et al. reported lower FVC value and FEV1 / FVC ratio [39]; Koc et al. reported lower FEV1 and FVC values in DM1 patients [40]. These results show that the lung function of DM1 patients isimpaired compared to the normal population. In addition, the PFT (FEV1, FVC) values of male patients in DM1 group were higher than those of female patients.

The positive correlation between PAV and DTai, DTae, FEV1, and FVC indicated that a regression analysis can be carried out. The amount of the PAV was mostly influenced by DTai and FVC. PAV was not significantly influenced by the DTae and FEV1 according to regression model. Further studies should be encouraged on the reasons of these different relations.

The conditions such as functional exercise capacity and inactivity of DM1 patients were not evaluated in the present study. Considering that this situation may affect the respiratory muscles and thus the airway volume, further studies can be planned on this subject. Another limitation of the present study was the difference of BMIs between the study groups. BMIs of the control group were significantly higher than those of the DM1 group. Bhatti et al. found that there is significant association between BMI and pulmonary function parameters. They stated that obesity has detrimental effects on respiratory system. Therefore, the results of the present study could have been affected by this situation [42].

The present study was the first study to use CBCT to evaluate the PAV of DM1 patients. In DM1 group, PAV was higher compared to control group. There was a significant positive correlation between the PAV values of DM1 patients and their diaphragm thickness and PFT values (*p <* 0.05). However, it was revealed that DTai and FVC had a considerable influence on PAV according to the regression analysis. It is recommended to evaluate the pharyngeal airway in DM1 patients because of impaired respiratory functions and pharyngeal muscle involvement.

## Figures and Tables

**Table 1:** Clinical parameters, diaphragm thickness, pulmonary function tests and pharyngeal airway volume.

	DM1 (N=30)	Control (N=30)	*p*
**Age**	42.40 ± 12.07	42.40 ± 12.07	1.000
**BMI**	24.56 ± 5.14	27.22 ± 3.51	**0.023** *
**PAV**	29082.60 ± 8407.63	18723.47 ± 3916.46	**<0.001** *
**AO**	22.37 ± 8.45		
**DD**	20.03 ± 12.08		
**DTai**	2.60 ± 0.65		
**DTae**	1.94 ± 0.40		
**FEV1**	2.77 ± 1.22		
**FVC**	3.33 ± 1.29		

The results are expressed as the mean ± standard deviation.

* significant differences between the groups. The independent samples *t*-test (^*^p < 0.05).

AO: Age of onset. BMI: Body mass index (kg/m^2^). DD: Disease duration. DM1: Myotonic dystrophy Type 1. DTae: Diaphragm thickness after expiration. DTai: Diaphragm thickness after inspiration. FEV1: Forced expiratory volume in 1 second. FVC: Forced volume vital capacity. PAV: Pharyngeal airway volume (mm^3^).

**Table 2:** Clinical parameters, diaphragm thickness, pulmonary function tests, and pharyngeal airway volume by sex.

DM1	Female (N=10)	Male (N=20)	*p*
**Age**	51 (25–69)	38.5 (19–57)	0.108
**AO**	20 (14–42)	18.5 (11–45)	0.675
**DD**	25.5 (10–45)	17.5 (2–38)	0.071
**BMI**	24.2 (20.2–34.1)	22.1 (18.7–30.8)	0.627
**DTai**	2.75 (1.7–3.4)	2.60 (1.5–4.3)	0.724
**DTae**	1.85 (1.1–2.6)	1.85 (1.2–2.8)	0.982
**FEV1**	1.06 (1.05–4.45)	3.13 (2.01–4.73)	**0.021** *
**FVC**	1.39 (1.38–5.03)	3.63 (2.76–5.93)	**0.012** *
**PAV**	22635 (13409–36418)	34065 (13561–43820)	**0.022** *
**Control**	**Female (N=10)**	**Male (N=20)**	***p***
**Age**	51 (25–69)	38.5 (19–57)	0.108
**BMI**	23.8 (19.4–28)	28.7 (20.5–32.1)	**0.001** *
**PAV**	18101 (11068–23883)	19127 (13372–27153)	0.538

The results are expressed as the median (minimum–maximum).

* indicate the significant differences between groups. The Mann–Whitney *U* test (^*^p < 0.05).

AO: Age of onset. BMI: Body mass index (kg/m^2^). DD: Disease duration. DM1: Myotonic dystrophy Type 1. DTae: Diaphragm thickness after expiration. DTai: Diaphragm thickness after inspiration. FEV1: Forced expiratory volume in 1 second. FVC: Forced volume vital capacity. PAV: Pharyngeal airway volume (mm^3^).

**Table 3:** Correlations between clinical parameters, diaphragm thickness, pulmonary function tests and PAV of DM1 patients

	AO	DD	BMI	DTai	DTae	FEV1	FVC	PAV
**DD**	−0.362*	1	0.469**	0.031	−0.209	−0.321	−0.394*	−0.305
*p*	**0.050**	.	**0.009**	0.870	0.269	0.083	**0.031**	0.101
**BMI**	0.034	0.469**	1	−0.083	−0.093	−0.279	−0.418*	−0.312
*p*	0.860	**0.009**	.	0.663	0.623	0.135	**0.021**	0.093
**DTai**	−0.194	0.031	−0.083	1	0.533**	0.122	0.154	0.681**
*p*	0.305	0.870	0.663	.	**0.002**	0.521	0.418	**<0.001**
**DTae**	−0.166	−0.209	−0.093	0.533**	1	0.673**	0.646**	0.679**
*p*	0.380	0.269	0.623	**0.002**	.	**<0.001**	**<0.001**	**<0.001**
**FEV1**	−0.386*	−0.321	−0.279	0.122	0.673**	1	0.766**	0.641*
*p*	0.035	0.083	0.135	0.521	**<0.001**	.	**<0.001**	**<0.001**
**FVC**	−0.298	−0.394*	−0.418*	0.154	0.646**	0.766**	1	0.831*
*p*	0.110	**0.031**	**0.021**	0.418	**<0.001**	**<0.001**	.	**<0.001**
**PAV**	−0.236	−0.305	−0.312	0.681**	0.679**	0.641*	0.831*	1
*p*	0.208	0.101	0.093	**<0.001**	**<0.001**	**<0.001**	**<0.001**	.

* Correlation is significant at the 0.05 level.

** Correlation is significant at the 0.01 level (Pearson and Spearman correlations, two-tailed).

AO: Age of onset. BMI: Body mass index (kg/m^2^). DD: Disease duration. DM1: Myotonic dystrophy Type 1. DTae: Diaphragm thickness after expiration. DTai: Diaphragm thickness after inspiration. FEV1: Forced expiratory volume in 1 second. FVC: Forced volume vital capacity. PAV: Pharyngeal airway volume (mm^3^).
